# Cannabidiol in Gliomas: Therapeutic Potential and Nanocarrier Strategies, with an Emphasis on Vesicular Delivery Systems

**DOI:** 10.1021/acs.molpharmaceut.5c00853

**Published:** 2025-11-25

**Authors:** Jagoda Szkudlarek, Ludwika Piwowarczyk, Anna Jelińska

**Affiliations:** † Chair and Department of Pharmaceutical Chemistry, 37807 Poznan University of Medical Sciences, 3 Rokietnicka, Poznań 60-806, Poland; ‡ Doctoral School, Poznan University of Medical Sciences, Bukowska 70, Poznań 60-812, Poland

**Keywords:** cancer, glioblastoma multiforme, GBM, cannabidiol, CBD, bioavailability, liposomes, vesicular systems

## Abstract

Cannabidiol (CBD), a nonpsychoactive phytocannabinoid extracted from *Cannabis sativa*, has emerged as a compound of considerable therapeutic interest across numerous medical disciplines, including pain management, anti-inflammatory therapy, and oncology. This review critically examines the potential of CBD in the treatment of glioblastoma multiforme (GBM), one of the most aggressive and treatment-resistant primary brain tumors. Particular emphasis is placed on the molecular mechanisms underlying CBD’s antitumor activity, including the modulation of key signaling pathways, inhibition of tumor proliferation, and enhancement of chemosensitivity. Furthermore, the review highlights the increasing role of nanotechnology in overcoming the intrinsic pharmacokinetic limitations of CBD, particularly its low oral bioavailability, which presents a significant challenge to its clinical application. Advanced nanocarrier platforms, including nanoemulsions, nanoparticles, nanoparticle-based transdermal systems, nanocapsules, and liposomes, have shown promise in optimizing CBD delivery to the central nervous system (CNS). Notably, the integration of CBD into lipid-based drug delivery systems (LBDDS) is highlighted as a particularly promising strategy to potentiate its therapeutic efficacy. This approach enhances bioavailability and may amplify synergistic effects when combined with conventional chemotherapeutics or targeted agents. Overall, the synergistic use of nanotechnological approaches and CBD-based therapies may open new avenues for research, offering the potential to significantly advance treatment efficacy in glioblastoma and other diseases.

## Graphical abstract

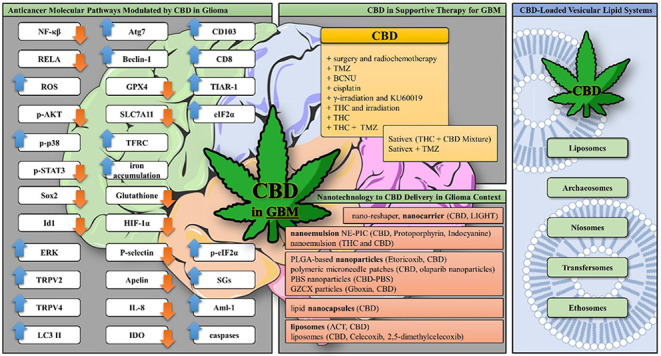

## 1 Introduction

Cannabis has been used medicinally since the 16th century in Asia for pain relief, reducing inflammation, and treating various conditions like seizures and insomnia. In 1840, William O’Shaughnessy highlighted its potential for ailments like asthma, sleep disorders, and opium withdrawal, paving the way for its therapeutic exploration. Over 560 compounds have been identified in cannabis, many with unique properties that could make them viable drug candidates.^1^ Many studies have investigated the anticancer effects of plant-derived natural compounds, utilizing both in vitro and in vivo models.^2^ Among these, CBD stands out as the second most abundant compound in Cannabis, with promising therapeutic effects, including sedation, anti-inflammation, antioxidation, and neuroprotection.^3,4^ CBD is notable in that it lacks psychoactive properties such as hallucinations and addiction, making it of growing interest.^5,6^ However, its medical use is limited by poor water solubility, low bioavailability, and unstable pharmacokinetics.^3,6^ Despite these challenges, CBD is widely consumed in unapproved over-the-counter products of unknown composition.^7^ The chemical formula of the CBD is shown in Figure 1.

**Figure 1:**
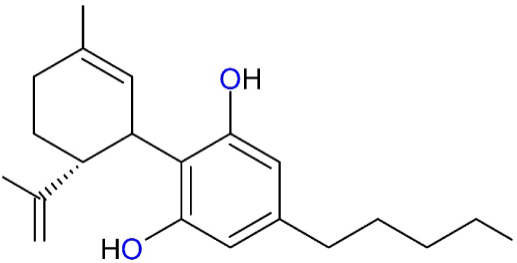
Chemical formula of CBD.^3^

CBD’s effects are believed to result from multiple molecular mechanisms involving G protein-coupled receptors (GPCRs) and ion channels.^4^ Additionally, CBD exhibits anticancer effects by interacting with the endocannabinoid system, leading to tumor cell inhibition, pain relief, and reduced chemotherapy-related side effects such as nausea and vomiting.^8^ Furthermore, CBD has the potential to enhance the effectiveness of conventional cancer therapies like chemotherapy and radiation while protecting against neurological and organ damage.^8,9^ Significant milestones in the medical use of CBD include the approval of Sativex and Epidiolex. A combination of two compounds found in *Cannabis sativa*, the psychotropic Δ^9‑^tetrahydrocannabinol (Δ^9‑^THC, THC) and CBD in Sativex (27 mg/mL Δ^9‑^THC; 25 mg/mL CBD), became the first CBD-based drug that the Medicines and Healthcare Products Regulatory Agency accepted for multiple sclerosis spasticity treatment (2010). Through an oromucosal spray, a significant portion of its CBD is absorbed through the gastrointestinal tract. Eight years later, Epidiolex (100 mg/mL CBD), the first drug with CBD as the sole active ingredient for treating rare epilepsy forms, was approved by the Food and Drug Administration.^10^

CBD has shown remarkable potential in inhibiting the progression of various cancers, including glioblastoma, lung, breast, colon, prostate, and melanoma, as demonstrated in numerous animal studies.^11^ However, CBD’s therapeutic potential is significantly impeded by its low oral bioavailability (about 6% in humans). Reasons for low bioavailability can include significant first-pass metabolism in the liver, instability in the acidic gastric pH, and poor water solubility. Interestingly, coadministration with a high-fat meal may increase CBD’s bioavailability by up to 4-fold.^12,13^ CBD’s high hydrophobicity, resulting in extremely low water solubility (approximately 2.3 × 10^–2^ μg/mL), further restricts its practical use.^14^ To overcome these challenges, advanced delivery systems are essential for improving the solubility and bioavailability of lipophilic substances, such as CBD.^15^

One promising approach to overcoming these limitations is nanotechnology, a rapidly advancing field that offers a promising, secure, cost-effective, and efficient approach to revolutionizing cancer treatment strategies.^16,18^ Nanoparticle-based drug delivery systems (DDS) enhance anticancer therapy by targeting drugs directly to cancer cells, reducing systemic toxicity, and improving therapeutic outcomes. Encapsulation protects drugs from degradation, ensures controlled release, and maximizes effectiveness while minimizing side effects.^19^ Nanoscale formulations have been widely studied to enhance lipophilic compounds’ solubility, release profiles, and bioavailability.^20^ Nanovesicles, a subcategory of lipid-based drug delivery systems (LBDDS), are widely used as carriers in DDS and stand out as one of the most prevalent choices.^21^ Among these, liposomes, a well-known representative of nanovesicles, are recognized as the first nanomedical delivery system to achieve clinical use and gain widespread acceptance.^22^

This review includes studies on the role of CBD in GBM. It proposes the use of CBD in LBDDS as a promising strategy to oppose GBM, addressing a significant gap in existing research. To our knowledge, no one has yet described or comprehensively summarized the research on the use of CBD in nanovesicles

## 2 Current Treatment Strategies and Challenges in GBM

Glioma, a type of primary brain tumor, includes GBM, the most common malignant tumor of the human CNS, and is characterized by a median survival of fewer than 15 months.^23,24^ The GBM’s aggressive nature is marked by pronounced heterogeneity and uncontrolled cell growth, significantly contributing to its poor prognosis.^24^ The intricate tumor microenvironment (TME) and its interaction with cancer cells significantly influence GBM growth and persistence.^25^ Adding to this challenge, glioma-initiating cells, known as GBM stem cells (GSCs), are among the most therapy-resistant cancer cells, further complicating treatment outcomes.^26^

Current therapeutic approaches, including surgery, radiotherapy, and chemotherapy with agents such as Temozolomide (TMZ), doxorubicin (DOXO), and carmustine (BCNU), have demonstrated only limited effectiveness in improving patient survival. Most GBM patients receive the Stupp regimen, the standard since 2005, which entails maximal safe resection; radiotherapy to 60 Gy in 2-Gy fractions, 5 days/week; concurrent TMZ 75 mg/m^2^; then up to six 28-day adjuvant cycles of TMZ 150–200 mg/m^2^ on days 1–5.^27^ Despite this standard approach, TMZ’s benefit is limited by resistance driven by tumor-intrinsic factors (such as MGMT expression and enhanced DNA-repair capacity) and microenvironmental influences (including hypoxia-induced signaling and immune evasion).^28^ Beyond surgery, RT, and chemotherapy, Tumor Treating Fields (TTFields) is a noninvasive, regional therapy for newly diagnosed GBM (with maintenance TMZ) and for recurrent GBM (monotherapy). Following the EF-14 trial, the U.S. FDA authorized TTFields after chemoradiation with maintenance TMZ. TTFields deliver low-intensity alternating electric fields that disrupt mitosis, improving progression-free and overall survival, although real-world adoption remains modest.^29,30^ Given GBM’s hyperemic biology with upregulated VEGFA and HIF, VEGFA is a rational target. Bevacizumab (BEV) is a humanized anti-VEGFA antibody with established activity in various cancers, including colorectal, cervical, renal cell carcinoma, and nonsquamous nonsmall cell lung cancer (NSCLC). Improves progression-free survival in GBM but has not demonstrated an overall-survival advantage. In recurrent GBM, combination regimens, particularly with lomustine and radiotherapy, outperform BEV monotherapy. Additionally, predictive factors for a better response include IDH mutation status, high tumor burden, and the double-positive sign.^31^ The dendritic cell (DC) vaccine is an emerging immunotherapy with supportive evidence for its use in treating glioblastoma. The Phase III DC-vaccine trial and meta-analysis showed that the DC vaccine, combined with standard care, was associated with significantly improved overall survival (HR = 0.71; 95% CI, 0.57–0.88) and progression-free survival (HR = 0.65; 95% CI, 0.43–0.98). In the subgroup of newly diagnosed glioblastoma patients, the DC vaccine was associated with improved progression-free survival (HR = 0.59; 95% CI, 0.39–0.90).^32^ The paucity of T cells limits the efficacy of immunotherapy in GBM within the tumor bed, resulting from both systemic and local immunosuppression. However, most research prioritizes the local immunosuppressive TME, overlooking the concurrent systemic immune suppression.^33^

Adoptive T-cell therapy with CAR-T cells remains a promising yet experimental approach in GBM. These engineered T cells target defined surface antigens and have transformed hematologic oncology since 2017; however, translation to solid tumors is limited by antigen heterogeneity and escape, suboptimal trafficking/penetration, and an immunosuppressive TME.^34^

Frustration with conventional therapies often drives patients with cancer to online sources touting cannabis benefits. They then trial heterogeneous products, ranging from whole-plant extracts to purified oils, and typically self-determine dosing. Numerous anecdotal favorable outcomes have been described, perpetuating interest in cannabis-based interventions.^35^

This highlights the urgent need to discover new therapies that enhance chemosensitivity and improve GMB patients’ results.^25,36^ The schematic of GBM selected characteristics is shown in Figure 2.

**Figure 2:**
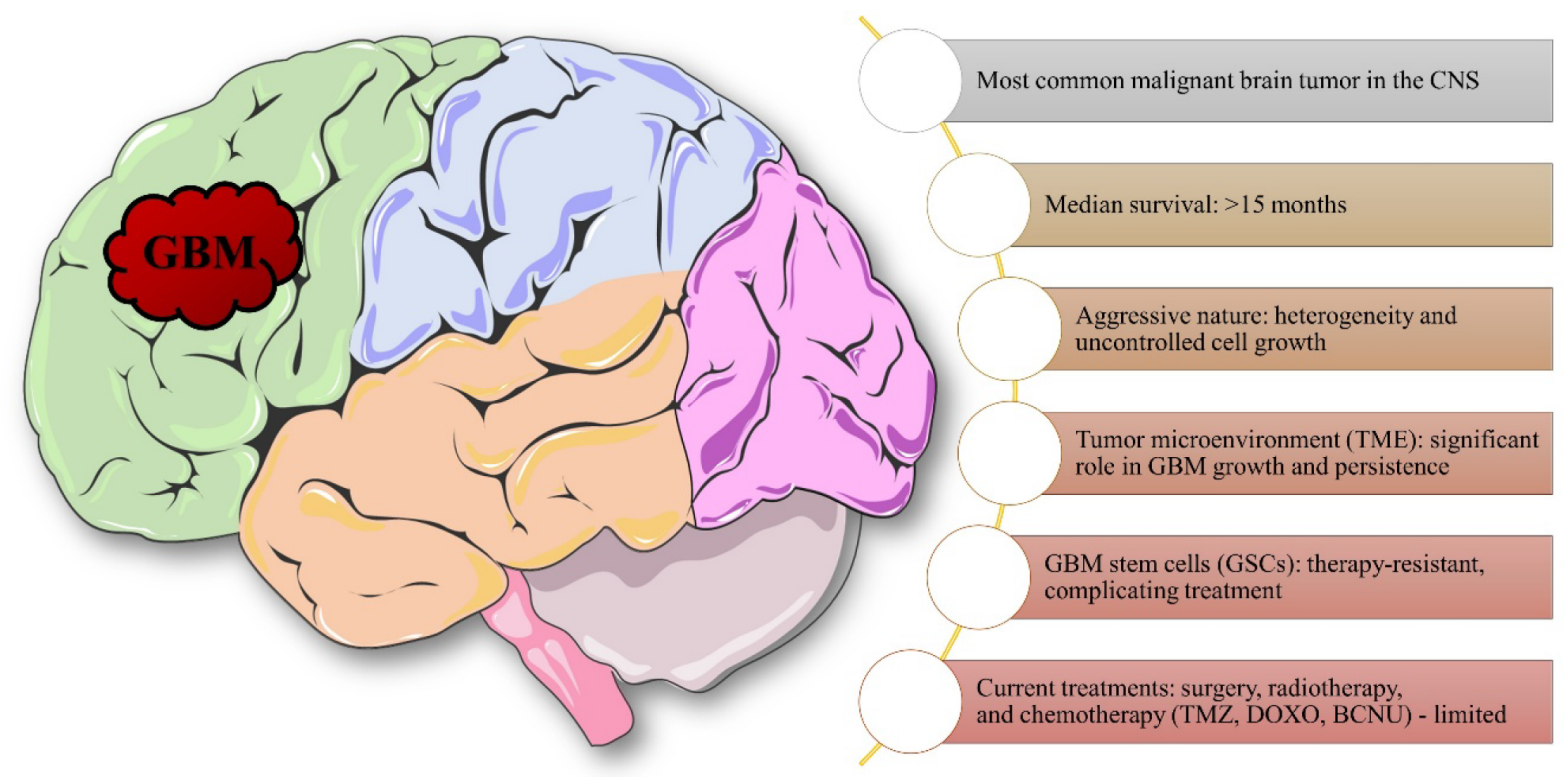
Schematic of GBM-selected characteristics.

## 3 Progress and Perspectives in CBD Studies on GBM

Despite promising pharmacology, cannabinoids’ clinical use is constrained by psychoactivity and marked lipophilicity. However, nonpsychoactive CBD has been shown to alleviate cancer-related symptoms and exert antitumor actions by inducing reactive oxygen species (ROS)-mediated apoptosis, modulating autophagy, regulating immune checkpoints, inhibiting angiogenesis, and reducing cell migration. Emerging evidence further indicates that CBD can sensitize tumors to standard therapies, enhancing the efficacy of chemotherapy and radiotherapy, and may act synergistically with existing treatments in malignant brain tumors.^37,38^ Given the therapeutic resistance of GBM and its persistently poor survival outcomes, plant-derived agents, notably CBD, warrant evaluation as adjuncts, or where appropriate, alternatives to current modalities. Prospective research and rigorously designed clinical trials are necessary to validate these observations, optimize delivery strategies (including nanomedicine formulations), and define the therapeutic role of CBD in glioma, with the aim of improving patient outcomes and survival.^39^

### 3.1 Anticancer Molecular Pathways Modulated by CBD in Glioma

At the molecular level, CBD’s impact on GBM is profound. The transcription factor NF-κB promotes cancer progression, while CBD can transform NF-κB into a tumor suppressor. Volmar et al. discovered that CBD enhances DNA binding of the NF-κB subunit v-rel avian reticuloendotheliosis viral oncogene homologue A (RELA) while inhibiting its phosphorylation at serine-311, a critical site for genetic transactivation. Additionally, CBD sensitivity was prominent in human primary GBM stem-like cells (hGSC) with low ROS levels. In contrast, high ROS levels in other tumors inhibited CBD-induced hGSC death, making ROS a predictive marker for CBD responsiveness.^37^ The transcriptional regulator Id-1 is present in GBM cell lines and primary cultures, and its expression is associated with heightened invasiveness. In U251 cells, CBD concentration-dependently reduced Id-1, which correlated with inhibition of cell invasion, with similar effects seen in primary GBM cells. CBD altered the phosphorylation of key kinases, including AKT, in U251 cells. CBD suppresses GBM invasion by organotypic brain slice and decreases Id-1 expression and GBM progression in vivo.^40^ Similarly, CBD increased ROS levels in GSCs, reducing cell survival, phosphorylated (p)-AKT, self-renewal, and enhancing survival in GSC-bearing mice. CBD-inhibited self-renewal was linked to p-p38 pathway activation and reduced pivotal stem cell regulators p-STAT3, Sox2, and Id1. However, some GSCs adapted, causing tumor regrowth. Combining CBD with system Xc (key in antioxidant response) inhibition increased ROS, significantly reducing GSC survival, self-renewal, and invasion.^41^ Kim et al. demonstrated that in GBM cells (U87, U373), CBD enhanced autophagy-related protein (LC3 II, Atg7, Beclin-1) and altered ferroptosis-related proteins (GPX4, SLC7A11, TFRC). CBD also raised endoplasmic reticulum (ER) stress, ROS levels, and iron accumulation while reducing Glutathione (GSH) levels.^42^ In vitro studies showed that CBD reduced U87-MG and T98G cell proliferation and invasiveness by downregulating proteins linked to growth, invasion, and angiogenesis. In U87-MG cells, CBD suppressed ERK and Akt prosurvival pathways in a dose-dependent manner and decreased hypoxia-inducible factor (HIF-1α) expression.^43^

The role of TRPV channels in mediating CBD’s effects has gained increasing attention. The activation of transient receptor potential vanilloid type 2 (TRPV2) has been demonstrated to reduce the growth of GBM cells and mitigate their resistance to chemotherapy agents. Nabissi et al. showed that CBD-induced TRPV2 activation affects glioma cell (U87MG cell line and primary glioblastoma cells from a patient with grade IV GBM) sensitivity to TMZ, BCNU, and DOXO. CBD enhances TRPV2 expression and activity, modulates Ca^2+^ influx, increases drug uptake, and synergizes with cytotoxic agents to apoptosis in glioma cells without affecting normal astrocytes.^36^ In another study, CBD upregulates acute myeloid leukemia (Aml-1) expression, crucial in GBM growth and differentiation, through a TRPV2 and PI3K/AKT-dependent mechanism. CBD-induced TRPV2 activation triggers autophagy, promoting Aml-1a-dependent GSC differentiation and overcoming BCNU resistance in GSCs.^44^ Huang et al. demonstrated that CBD-induced calcium flux via transient receptor potential cation channel subfamily V member 4 (TRPV4) activation was crucial for initiating mitophagy. High TRPV4 levels were linked to more aggressive tumors and poorer survival in patients with glioma. ER stress and the ATF4-DDIT3-TRIB3-AKT-MTOR axis (downstream of TRPV4) contribute to CBD-induced mitophagy.^23^

In another study, Lah et al. reported that CBD and phytocannabinoids’ precursor in the biosynthesis, cannabigerol (CBG), affect GBM, showing that both individually and combined induce caspase-dependent apoptosis. They identified GPR55 and TRPV1 receptors as key targets for CBD and CBG to remove GSCs. In patient-derived GSCs, the most effective cytotoxicity was observed with a 3:1 molar ratio of CBG to CBD.^26^

Marcu et al. demonstrated that CBD was more effective than Δ9-THC in inhibiting cell growth in SF126, U251, and U87 GBM cells, with lower IC_50_ values (CBD: 1.2, 0.6, and 0.6 μmol/L, respectively; Δ9-THC: 2.5, 3.3, and 3.3 μmol/L, respectively). Combined treatment of CBD with Δ9-THC significantly modulated the cell cycle, induced ROS and apoptosis, and specifically modulated ERK and caspase activities. Δ9-THC and CBD synergistically inhibited proliferation in U251 and SF126 cell lines, with unique effects on signal transduction pathways not seen with either compound alone.^45^ Adding a mechanistic perspective, Kolbe et al. investigated the effect of CBD and the GPR55 antagonist CID16020046 (CID) in effectively decreasing the reduction in Ki67 (a proliferation marker) caused by lysophosphatidylinositol (LPI), thereby confirming LPI’s binding to GPR55. They highlighted THC’s ability to bind to GPR55 and suggested that CBD may act as a potential antagonist at GPR55.^24^

Massi et al. conducted a study to assess the in vitro antiproliferative effects of CBD on U87 and U373 human glioma cell lines. CBD treatment led to a significant reduction in mitochondrial oxidative metabolism and cell viability, in a concentration-dependent manner, with an IC_50_ of 25 μM (after 24 h). This effect was partially mitigated by the CB2 receptor antagonist (SR144528; SR2) and alpha-tocopherol, but not by the CB1 receptor antagonist (SR141716; SR1), capsazepine, ceramide inhibitors, or pertussis toxin. CBD induced apoptosis, independent of cannabinoid receptor antagonists. In vivo, CBD administration (0.5 mg/mouse) significantly suppressed the growth of U87 glioma cells in nude mice.^46^ Turizo Smith et al. report that CBD, evaluated alongside Cannabichromene, cannabigerol, and *Piper nigrum* derivatives, shows high-affinity binding to glioblastoma-relevant targets GPR55 and PINK1. In in vitro assays, CBD exerted cytotoxic effects in glioblastoma cell lines (U87MG, T98G, CCF-STTG1), as well as in neuroblastoma (SH-SY5Y) and oligodendroglial (MO3.13) cells, with evidence of interactions among these compounds. The tumor-vs-normal differential expression of GPR55 and PINK1 further underscores their value as therapeutic targets and biomarkers.^39^

In studies with mice, the researchers highlight the potential of inhalation delivery of CBD for treating GBM. Khodadadi et al. exhibited that inhaled CBD inhibited GBM tumor growth and also modified the TME by reducing P-selectin, apelin, interleukin IL-8 and blocking indoleamine 2,3-dioxygenase (IDO), a key immune checkpoint. Additionally, CBD increased the expression of cluster of differentiation (CD) 103, suggesting enhanced antigen presentation, strengthened CD8 immune responses, and decreased the presence of innate Lymphoid Cells within the tumor.^25^ Wang et al. underscored that CBD treatment notably enhanced the volume of T-cell intracellular antigen-related proteins (TIAR-1), which was closely associated with an increase in eukaryotic translation initiation factor 2 alpha (eIF2α) expression and phosphorylation of eIF2α (*p*-eIF2α) in tissues treated with CBD compared to the placebo group (*p* < 0.05). Inhaled CBD markedly promoted the upregulation of stress granules (SGs) in GBM.^47^

CBD may modulate treatment responsiveness in cancer by altering the PI3K/AKT/mTOR and ERK signaling pathways. Studies indicate that combining CBD with standard chemotherapies can augment anticancer activity via these pathway effects.^35^

Translationally, these mechanisms map onto actionable biomarkers, RAD51 suppression, TRPV2 activity, NF-κB/STAT3 and Wnt/β-catenin signatures, that can guide patient selection and pharmacodynamic readouts in early phase GBM trials.

### 3.2 CBD in Supportive Therapy for GBM

In clinical settings, CBD has shown promising outcomes when used alongside standard GBM therapies. Likar et al. explored CBD as a supportive treatment for GBM alongside standard care (surgery and radiochemotherapy). In 2019, they demonstrated a case series of 9 brain tumor patients, including 6 with GBM, with longer survival rates with CBD (twice daily, dose: 100 mg, later usually increased to 200 mg).^48^ By 2021, the cohort expanded to 15 GBM patients, including the original 6. Patients received 400–600 mg of CBD daily, with two also taking 7.5 mg of THC. Among these patients, 46.7% (7 patients) survived for at least 24 months, and 26.7% (4 patients) lived for at least 36 months.^49^ In 2023, a follow-up of the same 15 GBM patients demonstrated a median survival of 28 months and an average of 30.9 months, which is 3–5 times longer than expected. Higher doses of CBD (400–600 mg/day) were linked to significantly longer survival, while lower doses (200 mg/day) corresponded with survival time less than the median value.^50^

One of the most compelling aspects of CBD research in GBM is its ability to enhance the efficacy of standard chemotherapies, particularly TMZ. TMZ resistance is often caused by methylguanine DNA-methyltransferase (MGMT) overexpression and DNA mismatch repair (MMR) deficiency. CBD and its derivative, 4′-Fluoro-cannabidiol (4′-F-CBD), which has demonstrated enhanced activity in vivo behavioral studies, have been shown to overcome these mechanisms in GBM. Brookes et al. identified methylated DNA as a previously unrecognized anticancer mechanism of CBD. Additionally, in vitro studies using human GBM cell lines U373–V (−MGMT, +MMR) and U373-M (+MGMT, +MMR) demonstrated synergy among imidazotetrazines (TMZ and its analog: T25) and cannabinoids (CBD and 4′-F-CBD).^51^ CBD improved TMZ’s effectiveness in U251 and U87MG cell lines and patient-derived GBM163 cells by increasing ROS levels, activating the ROS sensor AMP-activated protein kinase (AMPK), and elevating the autophagy marker LC3A. CBD sensitized U87 and GBM163 intracranial tumors to TMZ and showed a considerable increase in survival in tumor-bearing mice (the effect was absent in orthotopic GBM models with intact MGMT expression). CBD suppressed RAD51 expression in MGMT-methylated GBM models, suggesting a potential mechanism for its ability to enhance tumor sensitivity to TMZ.^52^ Deng et al. investigated that CBD dose-dependently reduced cell proliferation and viability across human GBM cell lines (T98G, U251, U87MG), PDGF-GBM cells from a mouse model, and cultured mouse neural progenitor cells (NPCs), exhibiting similar potency and lacking cancer-cell selectivity, through an allosteric mechanism in all tested cells. Combining CBD with DNA-damaging agents (TMZ, BCNU, cisplatin) resulted in synergistic antiproliferative and cytotoxic effects within a specific concentration range in the used cell lines. However, low concentrations caused antagonistic effects in some human and mouse GBM cells.^53^ Kosgodage et al. exhibited that CBD with TMZ increased antioncogenic miR126 while decreasing pro-oncogenic miR21 expression in GBM cells and GBM-derived extracellular vesicles (EVs), in comparison with treatment with only TMZ. CBD treatment reduced prohibitin (PHB), a protein involved in mitochondrial protection and chemoresistance in GBM cells.^54^ Additionally, combining CBD with TMZ synergistically inhibited tumor growth in patient-derived GBM cultures and glioma cell lines (U251, U87 MG, LN18, and GL261).^23^

In U87MG, U118MG, and T98G cell lines, CBD with γ-irradiation and KU60019 (ATM kinase inhibitor) increased apoptosis, G2/M cell cycle arrest, inhibited proliferation, and induced pro-inflammatory cytokine production, leading to apoptotic and inflammation-linked cell death. CBD-activated JNK-AP1, which has active NF-κB pathways, increased DR5/TRAIL-R2 gene and protein expression and enhanced GBM cell sensitivity to TRAIL-induced apoptosis. CBD significantly reduced PD-L1 surface levels in GBM cells, a key T-lymphocyte immune checkpoint. TS543 human proneural glioma neurospheres showed significant sensitivity to CBD-induced cell death, which was further enhanced when combined with γ-irradiation and KU60019.^55^

Scott et al. studied the effects of THC and CBD, alone and combined with radiotherapy, on human GBM cell lines (T98G, U87MG) and the mouse glioma cell line (GL261). Cannabinoids were tested as pure (P) and botanical drug substances (BDS). THC-BDS was more effective than THC-P, while CBD-P outperformed CBD-BDS. Pretreating cells with CBD-P and THC-P together preirradiation enhanced radiosensitivity compared to pretreating the cannabinoids individually. The enhanced radiosensitivity was linked with increased autophagy and apoptosis markers. In vivo, the combination of CBD, THC, and irradiation notably restrained tumor progression in an orthotopic syngeneic model.^56^ Using GBM cell lines (U87MG, U118MG, and T98G), scientists exhibited that CBD-mediated signaling can amplify γ-irradiation-induced cell death in GBM while having minimal impact on neural stem/progenitor cells and astrocytes. MAPK p38 was a major driver of CBD-induced cell death, whereas death levels after CBD + radiation treatment depended on MAPK p38 and JNK, which regulate endogenous TRAIL expression. CBD upregulated TNF/TNFR1 and TRAIL/TRAIL-R2 signaling (via ligand and receptor modulation) and apoptosis. NF-κB p65-P­(Ser536) was not a primary CBD target level of this factor, which was high in CBD-treated GBM cells, but its inhibition enhanced CBD-induced apoptosis.^57^

Scientists noticed that CBD significantly amplifies THC-induced autophagy, indicating its role in supporting THC’s autophagy activation pathway. CBD, in combination with THC, potentiates the antitumor effects of THC, thus potentially resulting in a reduced THC dose and a diminished incidence of psychoactive side effects in cannabinoid-based therapeutic regimens. Additionally, Sativex, currently used for palliative care in cancer and multiple sclerosis patients, alone or combined with TMZ, shows potential in managing GBM.^58^ López-Valero et al. found that combining oral administration of extracts containing equal amounts of THC and CBD (Sativex-like) with TMZ significantly reduced tumor growth in subcutaneous and intracranial U87MG glioma xenografts. However, combining Sativex-like extracts with BCNU offered no more significant benefit than using each treatment alone.^59^ In another scientific study, TMZ combined with THC:CBD mixtures (especially those with a higher CBD ratio but not TMZ combined with CBD only) achieved antitumor effects comparable to the THC:CBD (1:1 ratio) in U87MG glioma cell-derived xenografts. TMZ with a THC:CBD (1:1 ratio) inhibited the growth of orthotopic xenografts created with Glioma Initiating Cells (GICs) derived from GBM patient and improved survival in animals with these intracranial xenografts.^60^ Similarly, Torres et al. exhibited that coapplication of TMZ with submaximal doses of THC + CBD or a Sativex-like mixture (unlike CBD alone) significantly inhibited the growth of U87MG and T98G xenografts. Lower doses of THC + CBD enhanced TMZ’s antitumor effects and overcame glioma xenografts’ resistance to TMZ.^58^

### 3.3 Clinical Evidence for CBD in GBM

The current clinical evidence for CBD-containing interventions in GBM is limited but generates rationale for further study. In a randomized phase 1b study, adding a balanced THC:CBD oromucosal spray (nabiximols) to dose-intense TMZ at first recurrence yielded an acceptable safety profile and a signal for improved 12-month survival versus placebo, motivating confirmatory trials.^61^ A pivotal, biomarker-enriched, double-blind phase II trial (ARISTOCRAT) is underway to test nabiximols plus TMZ in recurrent MGMT-methylated GBM, with overall survival, progression-free survival, and HRQoL as key end points; its design directly addresses the limitations of earlier small studies.^62^ By contrast, a randomized trial of a high-CBD, full-spectrum hemp product in newly diagnosed GBM prioritized symptomatic end points and was terminated for low enrollment, underscoring operational hurdles and the need for oncology-centric outcomes.^63^ Prospective observational data with adjunct purified oral CBD suggest feasibility and reassuring tolerability with hypothesis-generating survival times, but causal inference is precluded by the nonrandomized design.^49^ A phase II study in high-grade glioma comparing fixed THC:CBD ratios showed good tolerability and improvements in selected patient-reported outcomes, with a 1:1 ratio favored—findings that inform supportive-care domains but are not themselves definitive for antitumor efficacy.^64^ Collectively, the evidence base is constrained by small sample sizes, heterogeneous formulations (purified CBD vs THC:CBD combinations), variable dosing/titration schemes, and inconsistent primary end points, which complicate cross-study comparisons. Systematic and narrative reviews converge on the conclusion that while preclinical anticancer activity of cannabinoids is plausible, robust clinical proof of efficacy in GBM remains unestablished.^65^ At the same time, mechanistic work identifies CBD-responsive pathways (e.g., modulation of NF-κB activity) that could guide biomarker-driven trials.^37^ Future studies should be adequately powered, stratified by molecular features (e.g., MGMT status, IDH mutation), and integrate PK/PD, CNS penetration, steroid exposure, and tumor-microenvironment readouts. Given challenges posed by the blood–brain barrier (BBB) and intratumoral heterogeneity, advanced delivery strategies (e.g., vesicular lipid systems and other nanoformulations of CBD) merit early phase clinical translation following encouraging preclinical data -provided that formulations, dosing algorithms, and clinically meaningful end points are standardized to enable rigorous efficacy testing within multimodal GBM care.^66^

Kenyon et al. emphasized nominating pharmaceutical-grade synthetic CBD as a potential therapy for breast cancer and glioma based on the evaluation of pharmaceutical-grade synthetic CBD (oily drops at 5% (w/v)) across 119 cancer patients (including 7 with GBM) using routinely collected data over four years. Clinical responses were observed in 92% of solid-tumor cases, as evidenced by reductions in circulating tumor cells and, in other patients, a decrease in tumor size. Additionally, they have no adverse effects.^35^

Table 1 summarizes the key clinical trials and cohort studies investigating CBD alone or THC:CBD combinations.

**Table 1:** Summary of Key Clinical Trials and Cohorts (CBD Alone or THC:CBD Combinations)

Study/Unit	Aim	Drug Formulation	Patient Group	Status	Main Conclusions	Dose/Schedule	Treatment Duration
Twelves et al. (Br J Cancer, 2021)^61^	Assess the safety and preliminary efficacy of adding nabiximols to dose-intense TMZ (DIT) at first GBM recurrence	Nabiximols (oromucosal spray; 1:1 THC:CBD) + DIT vs placebo + DIT	Adults with first recurrence GBM	Completed	Acceptable safety; 12-month OS 83% vs 44% (signal of efficacy); justified larger trial	Self-titrated to max 12 sprays/day (each 2.7 mg THC + 2.5 mg CBD); mean 7.5 sprays/day	Up to 12 months with TMZ per protocol
ARISTOCRAT Trial (UK) 2024 ^62^	Determine whether adding nabiximols to TMZ improves outcomes in recurrent MGMT-methylated GBM	Nabiximols (Sativex) + TMZ vs placebo + TMZ	Recurrent MGMT-methylated GBM	Ongoing (Phase II)	Double-blind, multicenter RCT; efficacy end points include OS/PFS and HRQoL	Self-titration starting at 1 spray nightly; min 3 and max 12 sprays/day; standard TMZ 5/28 days	Up to 6 months of nabiximols during maintenance TMZ
UCSF/NCT05753007 ^63^	Evaluate the impact of a high-CBD, full-spectrum hemp product on anxiety, QoL (and exploratory tumor outcomes) in newly diagnosed GBM on SOC	Full-spectrum, high-CBD oil (approximately 250 mg/mL CBD and 1.8 mg/mL THC) vs placebo	Newly diagnosed GBM during standard therapy	Terminated	Primary end points: anxiety, pain, QoL; tumor progression assessed exploratorily	Oral oil, blinded dosing (titrated as per protocol)	8 weeks double-blind treatment
Likar et al. (Cancer Diagnosis & Prognosis, 2021)^49^	Assess survival with adjunct oral CBD added to standard therapy	Purified oral CBD as comedication	15 adults with GBM (prospective cohort/observational)	Completed	Mean OS 24.2 mo (median 21); 47% ≥ 24 mo, 27% ≥ 36 mo; well tolerated (hypothesis-generating)	CBD 400–600 mg/day orally (added during/after SOC)	Variable; months to years in follow-up
Schloss et al. (Frontiers in Oncology, 2021)^64^	Tolerability of two THC:CBD ratios in high-grade glioma (subset GBM); QoL outcomes	THC:CBD oil (two ratios; oral, standardized medicinal cannabis)	High-grade glioma (incl. GBM)	Completed	Single nightly dose was safe/well tolerated; improved sleep and wellbeing; 1:1 ratio favored	Single nightly oral dose; two fixed THC:CBD ratios (1:1 and 4:1): 1:1—THC 4.6 mg/mL, CBD 4.8 mg/mL; 4:1—THC 15 mg/mL, CBD 3.8 mg/mL	12 weeks

### 3.4 Nanotechnology to CBD Delivery in Glioma Context

Nanotechnology has been identified as a promising solution to maximize the therapeutic potential of different substances and overcome the challenges associated with their delivery in GBM. In this context, CBD is particularly amenable to nanomedicine-based formulations.^38^ Currently, the number of studies using CBD in GBM using nanotechnology is limited, including using nanocarriers, nanoemulsions, nanoparticles (NPs), patches with NPs, nanocapsules, and liposomes.

Zhou et al. designed “Nano-reshaper,” a nanocarrier coencapsulating CBD (to alleviate lymphopenia) and the T-cell–recruiting cytokine LIGHT. Nanoreshaper expanded systemic T-cell pools and enhanced local T-cell recruitment, resulting in a marked increase in intratumoral T-cell infiltration. In GBM mouse models (males), combining Nanoreshaper with an immune checkpoint inhibitor yielded 83.3% long-term, recurrence-free survivors.^33^

Nanoemulsions, colloidal dispersions, can be used as drug carriers, mainly for molecules with a low solubility in water, composed of excipients of a safe quality.^67^ Borges et al. pointed to using multicharged nanoemulsions containing CBD in combination with photoactive agents. Tumorigenic (U87MG) and nontumorigenic (T98G) GBM cell lines demonstrated dose-dependent cytotoxic effects when exposed to the multicharged nanoemulsion NE-PIC (containing CBD, Protoporphyrin, and Indocyanine). As the CBD concentration in the nanoemulsion increased (from 0.5 to 4 mM), viability in both cell lines dropped significantly by more than 35% relative to untreated controls.^68^ Mobaleghol Eslam et al. developed a nanoemulsion containing the two drugs (NED) to enhance THC and CBD delivery in a rat C6 glioblastoma model. They benchmarked it against bulk drugs and a drug-free nanoemulsion (NE). The optimized NED measured 29 ± 6 nm, improving the hemocompatibility of the drugs. In vivo MRI and survival analyses revealed a ∼4-fold smaller tumor volume on day 7 compared to the control, and markedly prolonged survival: control, 9 days; bulk, 4 days; NE, 12.5 days; NED, 51 days.^69^

NPs with diameters less than 100 nm can be effective DDS, particularly for chemotherapy.^70^ Kuźmińska et al. developed PLGA-based NPs to codeliver Etoricoxib (ETO) and CBD, which significantly decreased cell (T98G, U-138 MG) viability in a dose-dependent manner and triggered apoptosis. The T98G cell line exhibited that ETO and CBD caused alterations in cell-cycle phase distribution following 24 h of incubation. CBD significantly alters cell-cycle distribution by increasing G1/G0 phase cells and decreasing those in S and G2/M phases in both lines (particularly at a 25 μM concentration; T98G cell line). Furthermore, intracellular accumulation of substances in NP-treated cancer cells was notably higher than treated with free substances, with CBD increasing 3.31-fold.^71^ In another study using NPs, Muresan et al. developed polymeric microneedle (MN) patches loaded with CBD or olaparib NPs, designed for insertion into brain resection cavities after tumor removal (isocitrate dehydrogenase wildtype glioblastoma), allowing the substances to diffuse up to 0.6 cm into brain tissue.^72^

Freire et al. showed that environmentally friendly PBS NPs loaded with CBD (CBD-PBS), but not empty PBS particles, produced a dose-dependent loss of viability in glioma lines (U118MG, U87MG). The formulation released ∼50% of CBD within hours, supporting controlled delivery and improved bioavailability. Both free and PBS-encapsulated CBD similarly decreased AKT Ser473 phosphorylation and increased LC3-II, confirming that nanoencapsulation preserves CBD’s signaling effects.^73^ Sun et al. developed GZCX (particles approximately 200 nm), a dual receptor–mediated, BBB-permeable carrier coloading Gboxin, a drug that was designed to inhibit the process of oxidative phosphorylation in GBM cells, and CBD for GBM treatment. GZCX achieved a 10-fold increase in brain Gboxin accumulation. In GBM-bearing mice, GZCX increased apoptosis, reduced angiogenesis, decreased tumor burden 3.7-fold, and prolonged survival. They emphasized the combined CLTX–CBD–mediated BBB penetration and GBM targeting, as well as receptor-mediated BBB transport by CLTX.^74^

In another work, researchers evaluated THC- and CBD-loaded poly-ε-caprolactone microparticles (particles approximately 50 μm) as a long-term delivery system in a murine glioma xenograft model. In vitro, microencapsulation enabled the sustained release of cannabinoids over several days. In vivo, in mice with glioma xenografts, local administration of THC, CBD, or a 1:1 (w/w) THC:CBD mix every 5 days suppressed tumor growth as effectively as daily local dosing of equivalent cannabinoid solutions. Treated tumors exhibited increased apoptosis, reduced proliferation, and decreased angiogenesis.^75^ Sharma et al. encapsulated CBD in magnesium-gallate metal–organic framework (MOF) microparticles (CBD/Mg-gallate-MOF), predominantly <2 μm. They achieved ∼65% CBD release at pH 7.2; this suppressed the viability of the rat glioma brain cancer (C6) cell line. CBD/Mg-GA exhibits anticancer activity by significantly increasing ROS and suppressing anti-inflammatory signaling, as evidenced by reduced TNF-α levels. Mechanistically, it modulates NF-κB, thereby initiating apoptotic cascades in glioma cells. In an in vitro BBB model, treatment reduced transendothelial electrical resistance (TEER) by 53.1%, indicating potential BBB penetration.^76^

Aparicio-Blanco et al. engineered PIT-made, monodisperse lipid nanocapsules (LNCs) decorated with nonpsychotropic cannabinoids and showed that the smallest constructs achieved the highest brain transcytosis. CBD conjugation enhances brain targeting 6-fold compared to G-Technology, the leading brain-active strategy already in clinical trials for CNS disorders.^77^ In U373MG assays, CBD-incorporated LNCs revealed strong size and surface effects: 20 nm LNCs lowered IC_50_ 3-fold versus 50 nm carriers; CBD decoration increased glioma targeting 3.4-fold; and combining CBD loading with CBD surface functionalization further reduced IC_50_.^38^

Another study demonstrated the potential of coloading ACT with selected phytochemicals (e.g., CBD) into cationic DOTAP:POPC liposomes. The nanoformulations were uniform and stable for 21 days, showing narrow size distributions, PDI < 0.3, and a negative zeta potential; vesicle diameters were <200 nm, supporting prospective clinical use, including GBM therapy. Encapsulation efficiency exceeded 84%. CBD-modulated liposomal molecular dynamics reduced molecular mobility and strengthened interaction networks, thereby adjusting particle size, whereas ACT alone retained the most excellent flexibility. Liposomes showed higher IC_50_ values in MRC-5 fibroblasts than in U-87 MG and U-138 MG glioma cells, indicating tumor-selective cytotoxicity; the ACT + CBD system achieved IC_50_ = 7 μM (U-87 MG) and 6 μM (U-138 MG) at 48 h, while the DOTAP:POPC control displayed higher IC_50_ and lower cytotoxicity. In glioma cells, ACT + CBD liposomes increased pro-apoptotic Bax and decreased antiapoptotic Bcl-xL in a dose- and time-dependent manner; the control had minimal effect.^78^ Rybarczyk et al. reported in a study using U-138 MG and T98G cell lines that CBD-, Celecoxib-, and 2,5-dimethylcelecoxib-containing liposomes exert robust anti-GBM effects by triggering apoptosis, oxidative stress, and modulating key signaling cascades. While coloading CBD with Celecoxib or 2,5-Dimethylcelecoxib did not yield clear synergistic or additive benefits, the CBD + Celecoxib combination suppressed Wnt/β-catenin and NF-κB signaling while activating the Nrf2 pathway.^79^

Table 2 provides a comprehensive summary of studies focused on the application of CBD formulations in glioma treatment. The table compares various formulations, models, and key outcomes, highlighting their effectiveness in improving CBD delivery, overcoming the blood-brain barrier, and enhancing therapeutic efficacy.

**Table 2:** Summarizing the Studies Focused on the Application of CBD Formulations in Glioma Treatment

Study	Aim	Formulations	Model	Key Outcomes
Zhou et al. (BMC Cancer 2023)^33^	Develop a nanostructure (Nanoreshaper) to coencapsulate CBD and the lymphocyte-recruiting cytokine LIGHT	Nanoreshaper (35 or 20 nm)—coencapsulates CBD and LIGHT	In vitro (GL261, GL261-luc, GL261-GFP, G422 cell lines), in vivo (mice bearing intracranial GL261 GBM)	Nanoreshaper expanded systemic T-cell pools and enhanced local T-cell recruitment, resulting in a marked increase in intratumoral T-cell infiltration. In GBM mouse models (males), combining Nanoreshaper with an immune checkpoint inhibitor yielded 83.3% long-term, recurrence-free survivors
Borges et al. (Photodiagnosis Photodyn. Ther. 2023)^68^	Design a nanoemulsion for CBD, indocyanine green (ICG), and protoporphyrin (PpIX) delivery in GBM treatment using photodynamic therapy (PDT)	Multicharged nanoemulsion NE-PIC (CBD, Protoporphyrin, and Indocyanine)	In vitro (U87MG, T98G cells)	U87MG and T98G GBM cell lines exhibited dose-dependent cytotoxicity when treated with NE-PIC. Increasing CBD concentration (0.5–4 mM) resulted in a decrease in cell viability of over 35% compared to controls
Mobaleghol et al. Eslam (BMC Pharmacol. Toxicol. 2024)^69^	Design a nanoemulsion (NE) to improve the delivery of THC and CBD from cannabis extracts in a glioblastoma animal model	Nanoemulsion with THC and CBD (NED) (29 ± 6 nm)	In vitro, in vivo (C6 glioma cells, rat model)	Increasing concentrations of CBD and THC reduced cell viability. NED reduced tumor volume (∼4-fold smaller) and significantly prolonged survival (51 days for NED vs 9 days for control)
Kuźmińska et al. (Pharmaceutics 2023)^71^	Explore the synergistic antitumor effects of CBD and etoricoxib (ETO) in a GBM cell line model, and develop poly(lactic-co-glycolic acid) (PLGA)-based NPs for the delivery of CBD and ETO	PLGA-based NPs with Etoricoxib (ETO) and CBD	In vitro (T98G, U-138 MG cells)	PLGA-based NPs with ETO and CBD decreased cell viability in a dose-dependent manner and triggered apoptosis. CBD alters cell-cycle distribution by increasing G1/G0 phase cells and decreasing those in S and G2/M phases. NP-treated cancer cells increased intracellular CBD accumulation 3.31-fold compared to free substances
Muresan et al. (Eur. J. Pharm. Biopharm. 2023)^72^	Created polymeric microneedle (MN) patches for placement in resection cavities after tumor surgical removal, such as in isocitrate dehydrogenase wild-type glioblastoma (GBM)	Polymeric microneedle (MN) patches with CBD or olaparib NPs	In vitro, in vivo (isocitrate dehydrogenase wild-type GBM)	Patches for insertion into brain resection cavities after tumor removal, allowing the substances to diffuse up to 0.6 cm into brain tissue
Freire et al. (J. Drug Deliv. Sci. Technol. 2024)^73^	Develop CBD-loaded Poly(butylene succinate) (PBS) NPs for in vitro cancer models	PBS NPs with CBD (CBD-PBS) (175 nm)	In vitro (U118MG, U87MG glioma lines)	CBD-PBS produced a dose-dependent loss of viability. The formulation released ∼50% of CBD within hours, supporting controlled delivery and improved bioavailability. Both free and PBS-encapsulated CBD decreased AKT Ser473 phosphorylation and increased LC3-II
Sun et al. (Chem. Eng. J. 2023)^74^	Develop GZCX as a dual receptor-mediated, BBB-permeable carrier coloading Gboxin, alongside CBD for GBM treatment	CLTX-conjugated Gboxin-encapsulated zein/CBD-based particles (GZCX) (∼200 nm)	In vitro, in vivo (GBM-bearing mice)	GZCX increased brain Gboxin accumulation by 10-fold and, in GBM-bearing mice, enhanced apoptosis, reduced angiogenesis, decreased tumor burden by 3.7-fold, and prolonged survival. It highlighted combined CLTX–CBD-mediated BBB penetration and GBM targeting via CLTX receptor-mediated transport
de la Ossa et al. (PLOS ONE 2013)^75^	Examine CBD- and THC-loaded poly-ε-caprolactone microparticles for sustained cannabinoid administration in a murine glioma xenograft model	CBD- and THC-loaded poly-ε-caprolactone microparticles (∼50 μm)	In vitro (U87MG glioma cells), In vivo (mouse xenograft model)	Microparticles enabled sustained cannabinoid release in vitro over several days. In vivo, local administration of THC, CBD, or a 1:1 THC:CBD mix every 5 days suppressed tumor growth as effectively as daily dosing, promoting apoptosis, reducing proliferation, and decreasing angiogenesis
Sharma et al. (J. Mater. Chem. B 2021)^76^	Investigate the potential of bioactive, microporous magnesium gallate MOF for the simultaneous delivery of gallic acid and CBD to cancer cells, aiming to produce synergistic anticancer effects	CBD in magnesium-gallate metal–organic framework (MOF) microparticles (CBD/Mg-gallate-MOF)	In vitro (rat glioma brain cancer (C6) cell line)	CBD/Mg-GA particles (<2 μm) released ∼65% CBD at pH 7.2, reducing the viability of C6 glioma cells, increasing ROS, suppressing TNF-α, and modulating NF-κB to trigger apoptosis. In an in vitro BBB model, treatment reduced transendothelial electrical resistance (TEER) by 53.1%, suggesting potential BBB penetration
Aparicio-Blanco et al. (Mol. Pharm. 2019)^77^	Develop lipid nanocapsules (LNCs) for brain-targeted CBD delivery after intravenous administration	Monodisperse lipid nanocapsules (LNCs) decorated with nonpsychoactive cannabinoids	In vitro, in vivo (hCMEC/D3 cells, mice)	nanocapsules enhanced brain transcytosis, with CBD conjugation improving brain targeting 6-fold compared to G-Technology
Aparicio-Blanco et al. (Eur. J. Pharm. Biopharm. 2019)^38^	Evaluate LNCs decorated and loaded with CBD for glioma treatment, and as extended-release carriers	CBD in lipid nanocapsules (LNCs)	In vitro (U373MG glioblastoma cells)	CBD-incorporated LNCs showed significant size and surface effects: 20 nm LNCs reduced IC_50_ by 3-fold compared to 50 nm carriers, CBD decoration enhanced glioma targeting by 3.4-fold, and CBD loading with surface functionalization lowered IC_50_
Szkudlarek et al. (Pharmaceutics 2025)^78^	Develop liposomal nanoformulations of acteoside (ACT) with CBD, or naringenin (NG) for glioma treatment	Co-loading ACT with CBD into cationic DOTAP:POPC liposomes (size <200 nm)	In vitro (U-87 MG, U-138 MG glioma cells)	CBD-modulated liposomes reduced molecular mobility, enhancing particle interactions. ACT + CBD liposomes showed reduced IC_50_ (7 μM for U-87 MG, 6 μM for U-138 MG), increased Bax, and decreased Bcl-xL in a dose- and time-dependent manner, with high encapsulation efficiency
Rybarczyk et al. (Pharmaceutics 2025)^79^	Develop liposomal formulations with CBD, celecoxib, and 2,5-dimethylcelecoxib, individually and in combination, to evaluate their anti-GBM effects	Liposomal nanoformulations with CBD, celecoxib, and 2,5-dimethylcelecoxib (size <185 nm)	In vitro (U-138 MG, T98G cell lines)	CBD-, Celecoxib-, and 2,5-dimethylcelecoxib-containing liposomes exert robust anti-GBM effects by triggering apoptosis, oxidative stress, and modulating key signaling cascades. CBD + Celecoxib suppressed Wnt/β-catenin and NF-κB signaling and activated the Nrf2 pathway

The CBD research in GBM, molecular mechanisms, role in supportive therapy, and nanotechnology-based delivery strategies are shown in Figure 3.

**Figure 3:**
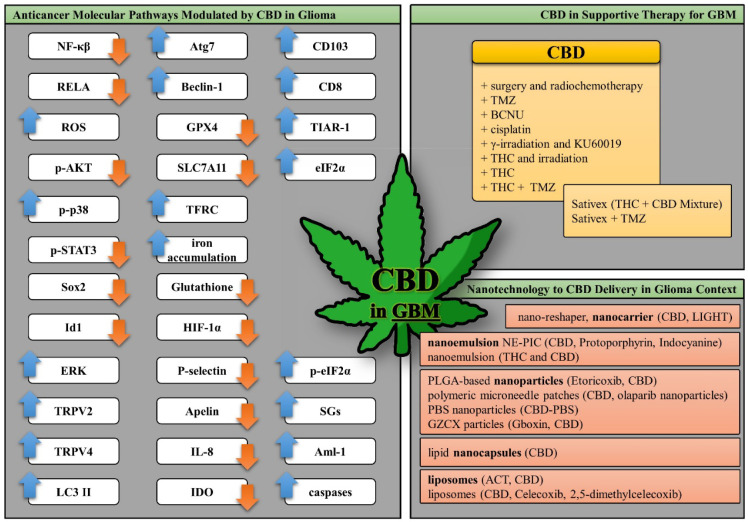
CBD research in GBM, molecular mechanisms, role in supportive therapy, and nanotechnology-based delivery strategies.

## 4 Perspectives on Vesicular Lipid Systems in GBM

Vesicular lipid carriers have a documented advantage in traversing or bypassing the BBB owing to their biophysical compatibility with endothelial lipid membranes, their capacity to exploit receptor-mediated transcytosis, and their efficiency via the intranasal “nose-to-brain” route.^80–83^ Recent reviews indicate that intranasally administered liposomes increase brain deposition and retention while partially circumventing the BBB, yielding higher CNS drug levels and potentially lower systemic exposure compared with conventional routes.^80,81^ In GBM models, intranasal liposomes have demonstrated direct distribution to brain and tumor tissue, with delivery further modulated by interventions affecting meningeal lymphatic function, providing strong evidence for the effectiveness of vesicular carriers in this pathway.^82^ High-impact evidence indicates that transferrin-targeted (and other ligand-modified) liposomes exploit receptor-mediated transcytosis in postcapillary venules, providing a demonstrable pathway for vesicular carriers to traverse the BBB.^83^ A critical translational proof comes from glutathione-PEGylated liposomal doxorubicin (2B3-101), which achieved approximately 5-fold higher brain penetration and prolonged survival in high-grade glioma models relative to conventional liposomes (Caelyx/Doxil).^84–86^ The same technology was subsequently tested clinically (phase 1/2a) in patients with primary and metastatic brain tumors, providing clinical feasibility evidence for BBB-targeted vesicular carriers.^85,86^ Niosomes, as a closely related vesicular class based on nonionic surfactants, have likewise shown enhanced brain uptake after intranasal administration (e.g., bromocriptine) together with favorable stability profiles, broadening the vesicular toolkit for CNS-directed agents such as CBD.^87,88^ In contrast to many nonvesicular platforms (polymeric or inorganic), vesicular lipid systems uniquely satisfy three criteria of direct relevance to GBM translation: demonstrated BBB transport via RMT, validated BBB bypass via the intranasal route, and clinical-class evidence (2B3-101), collectively yielding a favorable risk–benefit profile for further development.^80–86^ These foundations support the design of early phase CBD trials employing vesicular carriers with intranasal delivery and/or receptor targeting, embedding CNS PK/PD readouts and GBM molecular stratification (e.g., MGMT/IDH), alongside head-to-head comparisons with competing platforms within harmonized protocols.^80–86,88^

## 5 CBD-Loaded Vesicular Lipid Systems

Formulation studies demonstrate that the high lipophilicity of CBD (log *P* ≈ 6–7) promotes its preferential partitioning into the lipid bilayers of vesicular carriers.^89,90^ This physicochemical affinity underlies the high encapsulation efficiency and structural stability of these systems, rendering vesicular lipid carriers particularly advantageous for CBD delivery (Figure 4).

**Figure 4:**
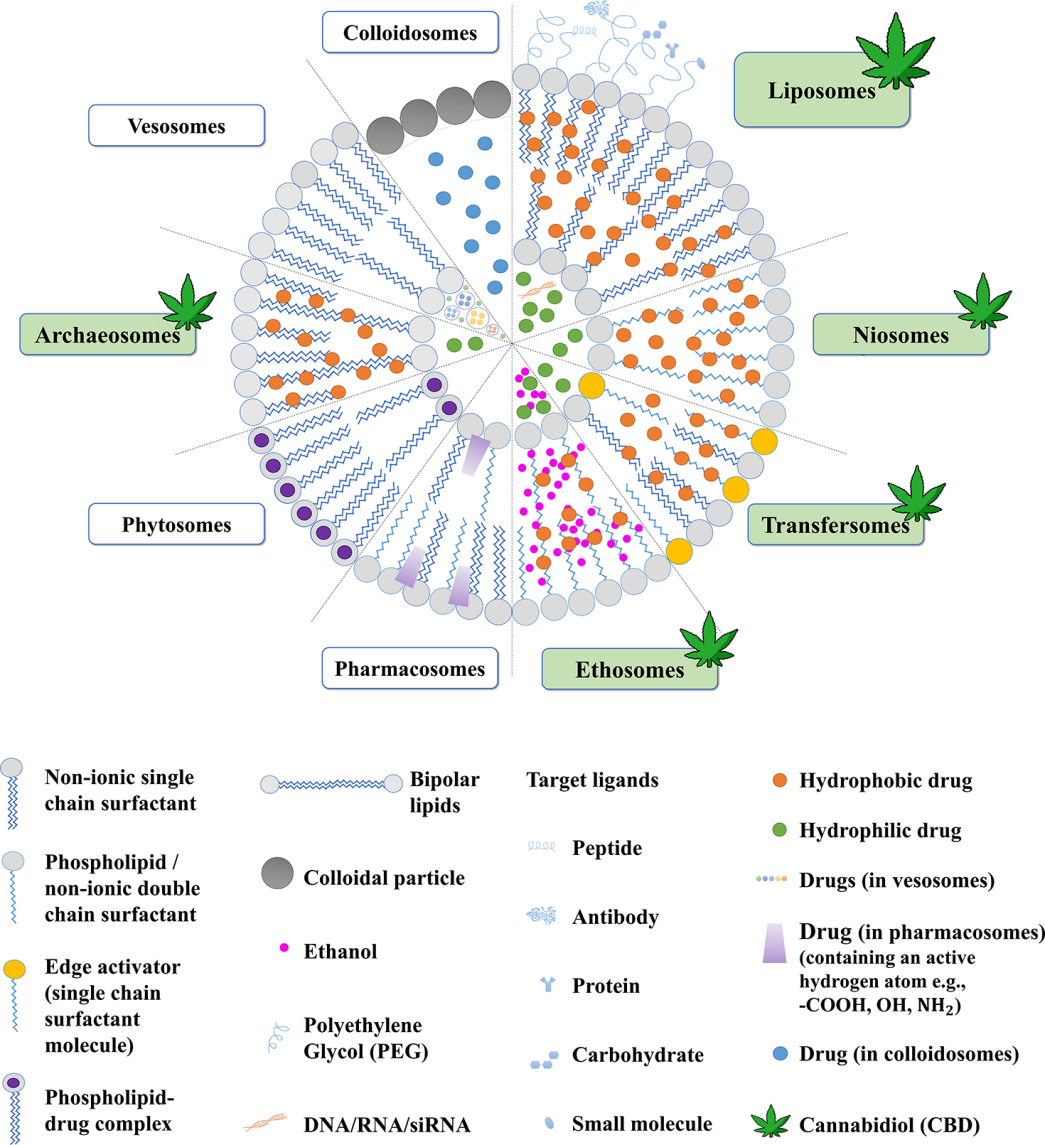
A schematic illustration of various vesicular systems (highlighting the vesicular systems used to deliver CBD).

Verrico et al. showed that CBD reduced proinflammatory cytokines IL-6 and TNF-α while increasing the anti-inflammatory IL-10 in vitro and mouse models. Liposomal formulation (∼100 nm liposomes encapsulating 10–20 mg/mL CBD) improved bioavailability in mice and humans and remained stable for three months (4 °C and pH 5–9).^91^ Blair and Miller demonstrated that THC-free liposomal hemp extract (containing 20% CBD and other cannabinoids) may help treat or reverse cancer-related cachexia and improve survival in a mouse model. Mice inoculated with colon 26 tumor cells and treated with 0.2 mg or 1 mg of THC-free liposomal hemp extract (additional doses to nonresponding or relapsing mice) showed improved weight gain and survival, with 4 of 7 mice receiving 1 mg and 2 of 7 receiving 0.2 mg surviving, compared to 1 of 9 control mice.^92^ Zapata et al. synthesized core–shell nanoliposomes (size <100 nm) containing CBD, which were noncytotoxic to nonmalignant human keratinocytes (HaCaT) cells up to 1000 mg·L^–1^, while higher concentrations exhibited antitumor activity against colon carcinoma (SW480) cells, suggesting potential as a chemotherapeutic agent.^93^ Jurgelane et al. prepared CBD-containing liposomes using commercial lipids DSPC, DPPC, and DSPE-PEG, with the following encapsulation efficiency: DPPC CBD (63%), DSPC CBD (74%), DSPC DPPC CBD (81%), DSPC DSPE-PEG CBD (87%). CBD release was highest initially in DPPC CBD liposomes, while DSPC DSPE-PEG CBD showed sustained release (79% over 504 h). The highest GMSC viability was observed at 96 h for all CBD liposomes, while liposomes without CBD showed no reduction in viability.^94^

Interestingly, in vitro experiments on bovine teeth suggested that olivetol-loaded γ-cyclodextrin metal–organic frameworks (γ-CD-MOFs) and DPPC liposomes are potential systems for delivering CBD in dental hypersensitivity treatment. Their findings revealed that olivetol, a precursor for CBD production, performed comparably to CBD, confirming its suitability as a CBD analog. The therapy reached the enamel and dentin layers.^95,96^

CBD has an impact on seizures and quality of life (QoL) in refractory frontal lobe epilepsy. Ebadi et al., conducted under a triple-blinding protocol, involved 27 patients (12 receiving a purified liposomal CBD preparation alongside standard medications, while 15 in the placebo group received only antiseizure drugs). After 4 weeks, 66.67% of CBD patients (versus 20.00% placebo) showed seizure improvement. Unlike standard ADEs, CBD significantly reduced seizure frequency by the study’s end time point [mean difference 45.58, 95% CI (8.987–82.18), *p* = 0.009]. At 8 weeks, QoLI-31­(quality of life questionnaire score) scores improved significantly from baseline [mean diff. −5.031, 95% CI (−9.729 to −0.333), *p* = 0.032], with a notable QoL improvement in the CBD group compared to placebo [RR 2.160, 95% CI (1.148 to 4.741), *p* = 0.018], though no significant difference was seen at 4 weeks (*p* = 0.653). QoL improvement was positively correlated with reduced seizure frequency [*r* = 0.638, 95% CI (0.296 to 0.835), *p* = 0.001], but in the CBD group alone, QoL changes were not associated with seizure severity or frequency (*p* > 0.05).^97^

Fu et al. explored the coencapsulation of CBD with 20­(S)-Protopanaxadiol (PPD), a ginseng-derived compound, to develop a low-toxicity antitumor agent. While neither CBD nor PPD alone showed strong antitumor effects, their coencapsulation in liposomes significantly inhibited tumor growth in 4T1 murine breast tumors, achieving an 82.2% inhibition rate (45 mg/kg i.v.), outperforming injection of 8 mg/kg of Paclitaxel (64.4%). CBD-PPD liposomes administered orally (45 mg/kg) resulted in a significantly lower TIR (56.8%) than those administered intravenously. The CBD-PPD liposomes (mean size 138.8 nm) caused no side effects, and the mice remained healthy and active.^98^

In topical applications, Franzè et al. investigated the potential of using deformable liposomes to deliver a combination of CBD and lidocaine (LD) into deeper skin. Two formulations were used: G-liposomes (CBD in the lipid bilayer and LD in the core via a pH gradient) and Drug-in-Micelles-in-Liposomes system (DiMiL), where LD was pre-encapsulated in micelles. DiMiL and G-liposomes outperformed control formulations with free drugs in enhancing skin penetration. The DiMiL enhanced skin permeation and dermal retention of substances compared to G-liposomes (CBD: 11.52 ± 2.4 vs 4.51 ± 0.8 μg/cm^2^; LD: 19.6 ± 2.9 vs 3.2 ± 0.1 μg/cm^2^). Moreover, removing Tween 80 from the liposome membrane, especially when a fluidizing agent like CBD is present, improves drug release and retention in the dermis.^99^ Additionally, Franzè et al. demonstrated that proliposomes, dry powders formed by coating phospholipids onto a porous, soluble carrier, serve as a promising intermediate for creating deformable liposome-based topical formulations, enhancing stability without affecting performance. The delivery of CBD through the human epidermis was significantly increased with DiMiL systems (developed by hydrating proliposomes with a micellar dispersion) compared to conventional deformable liposomes of identical lipid composition or an oil-based solution.^100^

Rao et al. introduced lipid nanocarrier bionic oleosomes (BOLE; natural lipid-based capsules with a triacylglycerol core encased by a dense monolayer of phospholipids and hydrophobic proteins) as an alternative to liposomes for encapsulating CBD. Using high-pressure homogenization, they created CBD-BOLE, demonstrating a 3.13 times greater loading efficiency and a 7.8 times higher CBD-phospholipid ratio than CBD-loaded liposomes. Moreover, CBD’s free radical scavenging capacity was enhanced while the cytotoxicity diminished.^14^ For cost-effective production of nanocarriers, Tiboni et al. indicated using 3D-printed polypropylene-based microfluidic devices produced via fused deposition modeling (FDM) printing. Microfluidic chips effectively produced liposomes and polymeric NPs loaded with CBD (as the model drug under experimental conditions) with adjustable properties and efficient drug loading.^101^

In addition to liposomes, the vesicular systems category encompasses niosomes, transfersomes, ethosomes, and archaeosomes, all of which have documented applications in CBD delivery, as well as pharmacosomes, phytosomes, vesosomes, and colloidosomes, which have not yet been explored in CBD-related research.

Ethosomes have exhibited significant promise in CBD transdermal delivery. Lodzki et al. highlight the promising potential of ethosomal carriers for transdermal CBD delivery, showcasing their ability to enhance skin permeation and create a localized CBD depot for targeted anti-inflammatory effects. In ICR mice, transdermal application of ethosomes to the abdomen maintained steady-state levels of CBD from 24 to 72 h. Moreover, ethosomal CBD by transdermal application effectively prevented inflammation and edema caused by carrageenan injection.^12^

Moqejwa et al. successfully created nanosized transferosomes loaded with CBD and formulated them into a rectal colloid to increase CBD absorption. The transferosomes, which exhibited a particle size distribution ranging from 102.2 to 130.1 nm, demonstrated encapsulation efficiency of 55.7–80.0% with different compositions of edge activators. Lyophilization ensured the preservation of their physicochemical properties for up to six months. *Ex vivo* studies indicated that, in comparison with pristine CBD, transferosomes significantly enhanced CBD diffusivity and permeation through excised colorectal membranes, while in vitro tests confirmed the trend and stable release kinetics of CBD.^5^

Gugleva et al. highlighted the potential of niosomes for CBD delivery. They demonstrated that amphiphilic copolymer-modified niosomal formulations effectively deliver CBD, maintaining its low micromolar cytotoxicity in tumor cells while enhancing its anti-inflammatory and pro-apoptotic properties. A formulation based on Tween 60:Span 60:Chol (3.5:3.5:3 molar ratio) with 2.5 mol % of a 3-arm star-shaped copolymer showed high CBD encapsulation efficiency (94%), a suitable particle size for parenteral use (235 nm), and controlled release. Encapsulated CBD formulations significantly enhance death receptor signaling (TRAILR2, FADD, Fas/CD95) and HIF-1α in *T*-24 urothelial cancer cells. Formulations with CBD reduce adhesion molecule ICAM-1, and CBD form of solution or in modified niosomes inhibits MMP-9 expression, likely lowering cancer invasiveness and metastasis. In mycosis fungoides CTCL-derived MJ cells, CBD and its niosomal form lower PON2 levels, reducing tolerance to free radicals in the inflammatory microenvironment. Additionally, they decrease cIAP-1 and survivin, potentially enhancing the chemosensitivity of the CTCL cells.^90^

The possibilities of archaeosomes for CBD delivery were demonstrated by Sedlmayr et al. in 2023, who evaluated archaeosomes (ether lipid-based) against lecithin-derived liposomes for delivering CBD orally. Archaeosomes showed a loading capacity six times higher than conventional liposomes and remained stable for more than six months after lyophilization. Under simulated GI tract conditions, CBD recovery was significantly higher in archaeosomes (57 ± 3%) than in conventional liposomes (34 ± 1%), while particle uptake in Caco-2 cells increased up to 6-fold. The researchers also suggested that archaeosomes are less dependent on the influence of food effects than conventional liposomes and commercial CBD oil products.^10^

## 6 Conclusion and Future Direction

CBD is a well-tolerated, nonpsychoactive phytocannabinoid that exhibits antitumor activity against GBM and glioma stem-like cells. This is achieved through mechanisms such as inducing oxidative stress, activating caspases, modulating ERK and TRPV2/TRPV4 signaling, and suppressing NF-κB and Id1. Convergent preclinical data and early clinical indications, particularly when used in conjunction with TMZ, support the need for rigorous clinical evaluation. Future work should prioritize well-controlled, biomarker-integrated clinical trials to refine CBD-based combination regimens within personalized treatment frameworks. In parallel, the systematic development of nanocarriers—particularly vesicular DDS—is required to enhance the therapeutic index of CBD by improving encapsulation, bioavailability, BBB transit, and tumor-targeted exposure.

## References

[1] Wang F., Multhoff G. (2021). Repurposing Cannabidiol as a Potential Drug Candidate
for Anti-Tumor Therapies. Biomolecules.

[2] Garcia-Oliveira P., Otero P., Pereira A. G., Chamorro F., Carpena M., Echave J., Fraga-Corral M., Simal-Gandara J., Prieto M. A. (2021). Status and Challenges of Plant-Anticancer
Compounds
in Cancer Treatment. Pharmaceuticals.

[3] Parlak
Khalily M. (2024). Improving the Water Solubility of Cannabidiol Using
a Peptide Carrier. Turk. J. Chem..

[4] de
Almeida D. L., Devi L. A. (2020). Diversity of Molecular Targets and
Signaling Pathways for CBD. Pharmacol. Res.
Perspect..

[5] Moqejwa T., Marimuthu T., Kondiah P. P. D., Choonara Y. E. (2022). Development of Stable
Nano-Sized Transfersomes as a Rectal Colloid for Enhanced Delivery
of Cannabidiol. Pharmaceutics.

[6] Grifoni L., Vanti G., Donato R., Sacco C., Bilia A. R. (2022). Promising
Nanocarriers to Enhance Solubility and Bioavailability of Cannabidiol
for a Plethora of Therapeutic Opportunities. Molecules.

[7] Britch S. C., Babalonis S., Walsh S. L. (2021). Cannabidiol: Pharmacology and Therapeutic
Targets. Psychopharmacology.

[8] Heider C. G., Itenberg S. A., Rao J., Ma H., Wu X. (2022). Mechanisms
of Cannabidiol (CBD) in Cancer Treatment: A Review. Biology.

[9] Mokoena D., George B. P., Abrahamse H. (2024). Cannabidiol Combination Enhances
Photodynamic Therapy Effects on MCF-7 Breast Cancer Cells. Cells.

[10] Sedlmayr V., Horn C., Wurm D. J., Spadiut O., Quehenberger J. (2023). Archaeosomes
Facilitate Storage and Oral Delivery of Cannabidiol. Int. J. Pharm..

[11] O’Brien K. (2022). Cannabidiol
(CBD) in Cancer Management. Cancers.

[12] Lodzki M., Godin B., Rakou L., Mechoulam R., Gallily R., Touitou E. (2003). Cannabidiol—Transdermal
Delivery
and Anti-Inflammatory Effect in a Murine Model. J. Controlled Release.

[13] Perucca E., Bialer M. (2020). Critical Aspects Affecting
Cannabidiol Oral Bioavailability
and Metabolic Elimination, and Related Clinical Implications. CNS Drugs.

[14] Rao Y., Tariq M., Wang M., Yu X., Liang H., Yuan Q. (2024). Preparation and Characterization of Bionics Oleosomes with High Loading
Efficiency: The Enhancement of Hydrophobic Space and the Effect of
Cholesterol. Food Chem..

[15] Onaivi E. S., Singh Chauhan B. P., Sharma V. (2020). Challenges of Cannabinoid Delivery:
How Can Nanomedicine Help?. Nanomedicine.

[16] Chaturvedi V. K., Singh A., Singh V. K., Singh M. P. (2019). Cancer Nanotechnology:
A New Revolution for Cancer Diagnosis and Therapy. Curr. Drug Metab..

[18] Dessale M., Mengistu G., Mengist H. M. (2022). Nanotechnology:
A Promising Approach
for Cancer Diagnosis, Therapeutics and Theragnosis. Int. J. Nanomed..

[19] Elumalai K., Srinivasan S., Shanmugam A. (2024). Review of the Efficacy of Nanoparticle-Based
Drug Delivery Systems for Cancer Treatment. Biomed. Technol..

[20] Stella B., Baratta F., Della Pepa C., Arpicco S., Gastaldi D., Dosio F. (2021). Cannabinoid Formulations
and Delivery Systems: Current and Future
Options to Treat Pain. Drugs.

[21] Sadeghi S., Ehsani P., Cohan R. A., Sardari S., Akbarzadeh I., Bakhshandeh H., Norouzian D. (2020). Design and Physicochemical Characterization
of Lysozyme Loaded Niosomal Formulations as a New Controlled Delivery
System. Pharm. Chem. J..

[22] Volmajer
Valh J., Peršin Z., Vončina B., Vrezner K., Tušek L., Fras Zemljič L. (2021). Microencapsulation
of Cannabidiol in Liposomes as Coating for Cellulose for Potential
Advanced Sanitary Material. Coatings.

[23] Huang T., Xu T., Wang Y., Zhou Y., Yu D., Wang Z., He L., Chen Z., Zhang Y., Davidson D., Dai Y., Hang C., Liu X., Yan C. (2021). Cannabidiol Inhibits
Human Glioma by Induction of Lethal Mitophagy through Activating TRPV4. Autophagy.

[24] Kolbe M. R., Hohmann T., Hohmann U., Ghadban C., Mackie K., Zöller C., Prell J., Illert J., Strauss C., Dehghani F. (2021). THC Reduces
Ki67-Immunoreactive Cells Derived from
Human Primary Glioblastoma in a GPR55-Dependent Manner. Cancers.

[25] Khodadadi H., Salles É. L., Alptekin A., Mehrabian D., Rutkowski M., Arbab A. S., Yeudall W. A., Yu J. C., Morgan J. C., Hess D. C., Vaibhav K., Dhandapani K. M., Baban B. (2023). Inhalant Cannabidiol Inhibits Glioblastoma Progression Through Regulation
of Tumor Microenvironment. Cannabis Cannabinoid
Res..

[26] Lah T. T., Majc B., Novak M., Sušnik A., Breznik B., Porčnik A., Bošnjak R., Sadikov A., Malavolta M., Halilčević S., Mlakar J., Zomer R. (2022). The Cytotoxic Effects of Cannabidiol
and Cannabigerol on Glioblastoma Stem Cells May Mostly Involve GPR55
and TRPV1 Signalling. Cancers.

[27] Rodgers L. T., Villano J. L., Hartz A. M. S., Bauer B. (2024). Glioblastoma Standard
of Care: Effects on Tumor Evolution and Reverse Translation in Preclinical
Models. Cancers.

[28] Jezierzański M., Nafalska N., Stopyra M., Furgoł T., Miciak M., Kabut J., Gisterek-Grocholska I. (2024). Temozolomide
(TMZ) in the Treatment of Glioblastoma Multiforme—A Literature
Review and Clinical Outcomes. Curr. Oncol..

[29] Khagi S., Kotecha R., Gatson N. T. N., Jeyapalan S., Abdullah H. I., Avgeropoulos N. G., Batzianouli E. T., Giladi M., Lustgarten L., Goldlust S. A. (2025). Recent Advances
in Tumor Treating Fields (TTFields) Therapy for Glioblastoma. Oncologist.

[30] Nabian N., Ghalehtaki R., Zeinalizadeh M., Balaña C., Jablonska P. A. (2024). State of the Neoadjuvant Therapy for Glioblastoma Multiforme—Where
Do We Stand?. Neuro-Oncol. Adv..

[31] Fu M., Zhou Z., Huang X., Chen Z., Zhang L., Zhang J., Hua W., Mao Y. (2023). Use of Bevacizumab
in Recurrent Glioblastoma: A Scoping Review and Evidence Map. BMC Cancer.

[32] Wong C.-E., Chang Y., Chen P.-W., Huang Y.-T., Chang Y.-C., Chiang C.-H., Wang L.-C., Lee P.-H., Huang C.-C., Hsu H.-J., Lee J.-S. (2024). Dendritic Cell Vaccine
for Glioblastoma:
An Updated Meta-Analysis and Trial Sequential Analysis. J. Neurooncol..

[33] Zhou S., Huang Y., Chen Y., Liu Y., Xie L., You Y., Tong S., Xu J., Jiang G., Song Q., Mei N., Ma F., Gao X., Chen H., Chen J. (2023). Reprogramming
Systemic and Local Immune Function to Empower Immunotherapy against
Glioblastoma. Nat. Commun..

[34] Park S., Maus M. V., Choi B. D. (2024). CAR-T Cell
Therapy for the Treatment
of Adult High-Grade Gliomas. NPJ. Precis Oncol..

[35] Kenyon J., Liu W., Dalgleish A. (2018). Report of
Objective Clinical Responses of Cancer Patients
to Pharmaceutical-Grade Synthetic Cannabidiol. Anticancer Res..

[36] Nabissi M., Morelli M. B., Santoni M., Santoni G. (2013). Triggering of the TRPV2
Channel by Cannabidiol Sensitizes Glioblastoma Cells to Cytotoxic
Chemotherapeutic Agents. Carcinogenesis.

[37] Volmar M. N. M., Cheng J., Alenezi H., Richter S., Haug A., Hassan Z., Goldberg M., Li Y., Hou M., Herold-Mende C., Maire C. L., Lamszus K., Flüh C., Held-Feindt J., Gargiulo G., Topping G. J., Schilling F., Saur D., Schneider G., Synowitz M., Schick J. A., Kälin R. E., Glass R. (2021). Cannabidiol Converts NF-κB
into a Tumor Suppressor in Glioblastoma with Defined Antioxidative
Properties. Neuro-Oncology.

[38] Aparicio-Blanco J., Sebastián V., Benoit J. P., Torres-Suárez A. I. (2019). Lipid Nanocapsules
Decorated and Loaded with Cannabidiol as Targeted Prolonged Release
Carriers for Glioma Therapy: In Vitro Screening of Critical Parameters. Eur. J. Pharm. Biopharm..

[39] Turizo
Smith A. D., Montoya Moreno N., Rodríguez-García J. A., Marín-Loaiza J. C., Arboleda Bustos G. (2025). Evaluating
the Antitumor Potential of Cannabichromene, Cannabigerol, and Related
Compounds from Cannabis sativa and Piper nigrum Against Malignant
Glioma: An In Silico to In Vitro Approach. Int.
J. Mol. Sci..

[40] Soroceanu L., Murase R., Limbad C., Singer E., Allison J., Adrados I., Kawamura R., Pakdel A., Fukuyo Y., Nguyen D., Khan S., Arauz R., Yount G. L., Moore D. H., Desprez P.-Y., McAllister S. D. (2013). Id-1 Is
a Key Transcriptional Regulator of Glioblastoma Aggressiveness and
a Novel Therapeutic Target. Cancer Res..

[41] Singer E., Judkins J., Salomonis N., Matlaf L., Soteropoulos P., McAllister S., Soroceanu L. (2015). Reactive Oxygen Species-Mediated
Therapeutic Response and Resistance in Glioblastoma. Cell Death Dis..

[42] Kim N. Y., Gowda S. G. S., Lee S.-G., Sethi G., Ahn K. S. (2024). Cannabidiol
Induces ERK Activation and ROS Production to Promote Autophagy and
Ferroptosis in Glioblastoma Cells. Chem. Biol.
Interact..

[43] Solinas M., Massi P., Cinquina V., Valenti M., Bolognini D., Gariboldi M., Monti E., Rubino T., Parolaro D. (2013). Cannabidiol,
a Non-Psychoactive Cannabinoid Compound Inhibits Proliferation and
Invasion in U87-MG and T98G Glioma Cells through a Multitarget Effect. PLoS One.

[44] Nabissi M., Morelli M. B., Amantini C., Liberati S., Santoni M., Ricci-Vitiani L., Pallini R., Santoni G. (2015). Cannabidiol
Stimulates
Aml-1a-Dependent Glial Differentiation and Inhibits Glioma Stem-like
Cells Proliferation by Inducing Autophagy in a TRPV2-Dependent Manner. Int. J. Cancer.

[45] Marcu J. P., Christian R. T., Lau D., Zielinski A. J., Horowitz M. P., Lee J., Pakdel A., Allison J., Limbad C., Moore D. H., Yount G. L., Desprez P.-Y., McAllister S. D. (2010). Cannabidiol Enhances the Inhibitory
Effects of Δ9-Tetrahydrocannabinol
on Human Glioblastoma Cell Proliferation and Survival. Mol. Cancer Ther..

[46] Massi P., Vaccani A., Ceruti S., Colombo A., Abbracchio M. P., Parolaro D. (2004). Antitumor Effects of
Cannabidiol, a Nonpsychoactive
Cannabinoid, on Human Glioma Cell Lines. J.
Pharmacol. Exp. Ther..

[47] Wang L. P., Chagas P. S., Salles É. L., Naeini S. E., Gouron J., Rogers H. M., Khodadadi H., Bhandari B., Alptekin A., Qin X., Vaibhav K., Costigliola V., Hess D. C., Dhandapani K. M., Arbab A. S., Rutkowski M. J., Yu J. C., Baban B. (2024). Altering Biomolecular
Condensates as a Potential Mechanism That Mediates Cannabidiol Effect
on Glioblastoma. Med. Oncol..

[48] Likar R., Koestenberger M., Stultschnig M., Nahler G. (2019). Concomitant Treatment
of Malignant Brain Tumours With CBD – A Case Series and Review
of the Literature. Anticancer Res..

[49] Likar R., Koestenberger M., Stutschnig M., Nahler G. (2021). Cannabidiol Μay
Prolong Survival in Patients With Glioblastoma Multiforme. Cancer Diagn. Progn..

[50] Likar R., Nahler G. (2023). Surprising Long Term
Survival in Glioblastoma Patients
Treated with Cannabidiol. Clin. Oncol. Res.

[51] Brookes A., Kindon N., Scurr D. J., Alexander M. R., Gershkovich P., Bradshaw T. D. (2024). Cannabidiol and
Fluorinated Derivative
Anti-Cancer Properties against Glioblastoma Multiforme Cell Lines,
and Synergy with Imidazotetrazine Agents. BJC
Rep..

[52] Soroceanu L., Singer E., Dighe P., Sidorov M., Limbad C., Rodriquez-Brotons A., Rix P., Woo R. W. L., Dickinson L., Desprez P.-Y., McAllister S. D. (2022). Cannabidiol
Inhibits RAD51 and Sensitizes
Glioblastoma to Temozolomide in Multiple Orthotopic Tumor Models. Neuro-Oncol. Adv..

[53] Deng L., Ng L., Ozawa T., Stella N. (2017). Quantitative Analyses of Synergistic
Responses between Cannabidiol and DNA-Damaging Agents on the Proliferation
and Viability of Glioblastoma and Neural Progenitor Cells in Culture. J. Pharmacol. Exp. Ther..

[54] Kosgodage U. S., Uysal-Onganer P., MacLatchy A., Mould R., Nunn A. V., Guy G. W., Kraev I., Chatterton N. P., Thomas E. L., Inal J. M., Bell J. D., Lange S. (2019). Cannabidiol
Affects Extracellular Vesicle Release, miR21 and miR126, and Reduces
Prohibitin Protein in Glioblastoma Multiforme Cells. Transl. Oncol..

[55] Ivanov V. N., Wu J., Wang T. J. C., Hei T. K. (2019). Inhibition of ATM Kinase Upregulates
Levels of Cell Death Induced by Cannabidiol and γ-Irradiation
in Human Glioblastoma Cells. Oncotarget.

[56] Scott K. A., Dalgleish A. G., Liu W. M. (2014). The Combination of Cannabidiol and
Δ9-Tetrahydrocannabinol Enhances the Anticancer Effects of Radiation
in an Orthotopic Murine Glioma Model. Mol. Cancer
Ther..

[57] Ivanov V. N., Wu J., Hei T. K. (2017). Regulation of Human
Glioblastoma Cell Death by Combined
Treatment of Cannabidiol, γ-Radiation and Small Molecule Inhibitors
of Cell Signaling Pathways. Oncotarget.

[58] Torres S., Lorente M., Rodríguez-Fornés F., Hernández-Tiedra S., Salazar M., García-Taboada E., Barcia J., Guzmán M., Velasco G. (2011). A Combined Preclinical
Therapy of Cannabinoids and Temozolomide against Glioma. Mol. Cancer Ther..

[59] López-Valero I., Torres S., Salazar-Roa M., García-Taboada E., Hernández-Tiedra S., Guzmán M., Sepúlveda J. M., Velasco G., Lorente M. (2018). Optimization
of a Preclinical
Therapy of Cannabinoids in Combination with Temozolomide against Glioma. Biochem. Pharmacol..

[60] López-Valero I., Saiz-Ladera C., Torres S., Hernández-Tiedra S., García-Taboada E., Rodríguez-Fornés F., Barba M., Dávila D., Salvador-Tormo N., Guzmán M., Sepúlveda J. M., Sánchez-Gómez P., Lorente M., Velasco G. (2018). Targeting Glioma Initiating Cells
with A Combined Therapy of Cannabinoids and Temozolomide. Biochem. Pharmacol..

[61] Twelves C., Sabel M., Checketts D., Miller S., Tayo B., Jove M., Brazil L., Short S. C. (2021). GWCA1208 study group.
A Phase 1b Randomised, Placebo-Controlled Trial of Nabiximols Cannabinoid
Oromucosal Spray with Temozolomide in Patients with Recurrent Glioblastoma. Br. J. Cancer.

[62] Bhaskaran D., Savage J., Patel A., Collinson F., Mant R., Boele F., Brazil L., Meade S., Buckle P., Lax S., Billingham L., Short S. C. (2024). A Randomised Phase II Trial of Temozolomide with or
without Cannabinoids in Patients with Recurrent Glioblastoma (ARISTOCRAT):
Protocol for a Multi-Centre, Double-Blind, Placebo-Controlled Trial. BMC Cancer.

[63] Gruber, S. A. Randomized, Double-Blind, Clinical Trial of a Hemp-Derived, High Cannabidiol Product for Anxiety in Glioblastoma Patients; Clinical trial registration NCT05753007; clinicaltrials.gov, 2025. https://clinicaltrials.gov/study/NCT05753007 (accessed 20 October 2025).

[64] Schloss J., Lacey J., Sinclair J., Steel A., Sughrue M., Sibbritt D., Teo C. (2021). A Phase 2 Randomised Clinical Trial
Assessing the Tolerability of Two Different Ratios of Medicinal Cannabis
in Patients With High Grade Gliomas. Front.
Oncol..

[65] Guggisberg J., Schumacher M., Gilmore G., Zylla D. M. (2022). Cannabis as an Anticancer
Agent: A Review of Clinical Data and Assessment of Case Reports. Cannabis Cannabinoid Res..

[66] Feng S., Pan Y., Lu P., Li N., Zhu W., Hao Z. (2024). From Bench
to Bedside: The Application of Cannabidiol in Glioma. J. Transl. Med..

[67] Sánchez-López E., Guerra M., Dias-Ferreira J., Lopez-Machado A., Ettcheto M., Cano A., Espina M., Camins A., Garcia M. L., Souto E. B. (2019). Current Applications
of Nanoemulsions
in Cancer Therapeutics. Nanomaterials.

[68] Borges H. S., Gusmão L. A., Tedesco A. C. (2023). Multi-Charged Nanoemulsion for Photodynamic
Treatment of Glioblastoma Cell Line in 2D and 3D in Vitro Models. Photodiagn. Photodyn. Ther..

[69] Mobaleghol
Eslam H., Hataminia F., Esmaeili F., Salami S. A., Ghanbari H., Amani A. (2024). Preparation of a Nanoemulsion Containing
Active Ingredients of Cannabis Extract and Its Application for Glioblastoma:
In Vitro and in Vivo Studies. BMC Pharmacol.
Toxicol..

[70] Zhang J., Lan C. Q., Post M., Simard B., Deslandes Y., Hsieh T. H. (2006). Design of Nanoparticles as Drug Carriers for Cancer
Therapy. Cancer Genomics Proteomics.

[71] Kuźmińska J., Sobczak A., Majchrzak-Celińska A., Żółnowska I., Gostyńska A., Jadach B., Krajka-Kuźniak V., Jelińska A., Stawny M. (2023). Etoricoxib-Cannabidiol Combo: Potential
Role in Glioblastoma Treatment and Development of PLGA-Based Nanoparticles. Pharmaceutics.

[72] Muresan P., McCrorie P., Smith F., Vasey C., Taresco V., Scurr D. J., Kern S., Smith S., Gershkovich P., Rahman R., Marlow M. (2023). Development of Nanoparticle
Loaded
Microneedles for Drug Delivery to a Brain Tumour Resection Site. Eur. J. Pharm. Biopharm..

[73] Freire N. F., Cordani M., Aparicio-Blanco J., Fraguas Sanchez A. I., Dutra L., Pinto M. C. C., Zarrabi A., Pinto J. C., Velasco G., Fialho R. (2024). Preparation and Characterization
of PBS (Polybutylene Succinate) Nanoparticles Containing Cannabidiol
(CBD) for Anticancer Application. J. Drug Delivery
Sci. Technol..

[74] Sun Y., Kong J., Ge X., Mao M., Yu H., Liu J., Wang Y. (2023). A Dual Receptor Targeting
and Blood–Brain Barrier
Penetrating Co-Drug-Loaded Particle Mediating Inhibition of Oxidative
Phosphorylation for Targeted Therapy of Glioblastoma. Chem. Eng. J..

[75] de
la Ossa D. H. P., Lorente M., Gil-Alegre M. E., Torres S., García-Taboada E., Aberturas M. D. R., Molpeceres J., Velasco G., Torres-Suárez A. I. (2013). Local Delivery
of Cannabinoid-Loaded Microparticles Inhibits Tumor Growth in a Murine
Xenograft Model of Glioblastoma Multiforme. PLoS One.

[76] Sharma A., Kumar A., Li C., Panwar Hazari P., Mahajan S. D., Aalinkeel R., Sharma R. K., Swihart M. T. (2021). A Cannabidiol-Loaded
Mg-Gallate Metal-Organic Framework-Based Potential Therapeutic for
Glioblastomas. J. Mater. Chem. B.

[77] Aparicio-Blanco J., Romero I. A., Male D. K., Slowing K., García-García L., Torres-Suárez A. I. (2019). Cannabidiol Enhances the Passage
of Lipid Nanocapsules across the Blood–Brain Barrier Both in
Vitro and in Vivo. Mol. Pharmaceutics.

[78] Szkudlarek J., Piwowarczyk L., Krajka-Kuźniak V., Majchrzak-Celińska A., Tomczak S., Baranowski M., Pietrzyk R., Woźniak-Braszak A., Jelińska A. (2025). Liposomal Co-Delivery of Acteoside, CBD, and Naringenin:
A Synergistic Strategy Against Gliomas. Pharmaceutics.

[79] Rybarczyk A., Majchrzak-Celińska A., Piwowarczyk L., Krajka-Kuźniak V. (2025). The Anti-Glioblastoma
Effects of
Novel Liposomal Formulations Loaded with Cannabidio, Celecoxib, and
2,5-Dimethylcelecoxib. Pharmaceutics.

[80] Duong V.-A., Nguyen T.-T.-L., Maeng H.-J. (2023). Recent
Advances in Intranasal Liposomes
for Drug, Gene, and Vaccine Delivery. Pharmaceutics.

[81] Koo J., Lim C., Oh K. T. (2024). Recent
Advances in Intranasal Administration for Brain-Targeting
Delivery: A Comprehensive Review of Lipid-Based Nanoparticles and
Stimuli-Responsive Gel Formulations. Int. J.
Nanomed..

[82] Semyachkina-Glushkovskaya O., Shirokov A., Blokhina I., Telnova V., Vodovozova E., Alekseeva A., Boldyrev I., Fedosov I., Dubrovsky A., Khorovodov A., Terskov A., Evsukova A., Elovenko D., Adushkina V., Tzoy M., Agranovich I., Kurths J., Rafailov E. (2023). Intranasal Delivery of Liposomes
to Glioblastoma by Photostimulation of the Lymphatic System. Pharmaceutics.

[83] Wu D., Chen Q., Chen X., Han F., Chen Z., Wang Y. (2023). The Blood–Brain Barrier: Structure, Regulation and Drug Delivery. Signal Transduction Targeted Ther..

[84] Gaillard P. J., Appeldoorn C. C. M., Dorland R., van Kregten J., Manca F., Vugts D. J., Windhorst B., van Dongen G. A. M. S., de Vries H. E., Maussang D., van Tellingen O. P. (2014). Pharmacokinetics,
Brain Delivery, and Efficacy in Brain Tumor-Bearing Mice of Glutathione
Pegylated Liposomal Doxorubicin (2B3–101). PLoS One.

[85] Kerklaan B. M., Jager A., Aftimos P., Dieras V., Altintas S., Anders C., Arnedos M., Gelderblom H., Soetekouw P., Gladdines W. (2014). NT-23: Phase 1/2A Study
of glutathione pegylated liposomal doxorubicin (2B3-101) In breast
cancer patients with brain metastases (BCBM) Or recurrent high grade
gliomas (HGG). Neuro-Oncology.

[86] Brandsma D., Dieras V., Altintas S., Anders C., Arnedos M., Gelderblom H., Soetekouw P., Jager A., van Linde M., Aftimos P. (2014). P08.03 2B3-101, Glutathione
pegylated liposomal doxorubicin,
in patients with recurrent high grade gliomas and breast cancer brain
metastases. Neuro-Oncology.

[87] Sita V. G., Jadhav D., Vavia P. (2020). Niosomes for Nose-to-Brain
Delivery
of Bromocriptine: Formulation Development, Efficacy Evaluation and
Toxicity Profiling. J. Drug Delivery Sci. Technol..

[88] Rinaldi F., Hanieh P. N., Chan L. K. N., Angeloni L., Passeri D., Rossi M., Wang J. T.-W., Imbriano A., Carafa M., Marianecci C. (2018). Chitosan Glutamate-Coated
Niosomes: A Proposal for
Nose-to-Brain Delivery. Pharmaceutics.

[89] Lacerda M., Carona A., Castanheira S., Falcão A., Bicker J., Fortuna A. (2025). Pharmacokinetics of
Non-Psychotropic
Phytocannabinoids. Pharmaceutics.

[90] Gugleva V., Ahchiyska K., Georgieva D., Mihaylova R., Konstantinov S., Dimitrov E., Toncheva-Moncheva N., Rangelov S., Forys A., Trzebicka B., Momekova D. D. (2023). Characterization and Pharmacological
Evaluation of
Cannabidiol-Loaded Long Circulating Niosomes. Pharmaceutics.

[91] Verrico C. D., Wesson S., Konduri V., Hofferek C. J., Vazquez-Perez J., Blair E., Dunner K., Salimpour P., Decker W. K., Halpert M. M. A. R. D.-B. (2020). Placebo-Controlled Study of Daily
Cannabidiol for the Treatment of Canine Osteoarthritis Pain. Pain.

[92] Blair E., Miller A. L. (2023). Liposomal Hemp Extract
for the Management of Cachexia. Bioact. Compd.
Health Dis..

[93] Zapata K., Rosales S., Rios A., Rojano B., Toro-Mendoza J., Riazi M., Franco C. A., Cortés F. B. (2023). Nanoliposomes
for Controlled Release of Cannabinodiol at Relevant Gastrointestinal
Conditions. ACS Omega.

[94] Jurgelane I., Egle K., Grava A., Galkina D., Brante M., Melnichuks M., Skrinda-Melne M., Salms G., Dubnika A. (2025). Exploring
the Effects of Cannabidiol Encapsulation in Liposomes on Their Physicochemical
Properties and Biocompatibility. Drug Delivery.

[95] Rodríguez-Martínez J., Sánchez-Martín M.-J., Valiente M. (2023). Efficient Controlled
Release of Cannabinoids Loaded in γ-CD-MOFs and DPPC Liposomes
as Novel Delivery Systems in Oral Health. Mikrochim.
Acta.

[96] Lago-Fernandez, A. ; Redondo, V. ; Hernandez-Folgado, L. ; Figuerola-Asencio, L. ; Jagerovic, N. Chapter Eleven - New Methods for the Synthesis of Cannabidiol Derivatives. In Methods in Enzymology; Cannabinoids and Their Receptors; Reggio, P. H. eds.; Academic Press, 2017; Vol. 593, pp. 237–257; 10.1016/bs.mie.2017.05.006. 28750806

[97] Ebadi S. R., Saleki K., Adl Parvar T., Rahimi N., Aghamollaii V., Ranji S., Tafakhori A. (2023). The Effect
of Cannabidiol on Seizure
Features and Quality of Life in Drug-Resistant Frontal Lobe Epilepsy
Patients: A Triple-Blind Controlled Trial. Front.
Neurol..

[98] Fu J., Zhang K., Lu L., Li M., Han M., Guo Y., Wang X. (2022). Improved Therapeutic
Efficacy of CBD with Good Tolerance
in the Treatment of Breast Cancer through Nanoencapsulation and in
Combination with 20­(S)-Protopanaxadiol (PPD). Pharmaceutics.

[99] Franzè S., Angelo L., Casiraghi A., Minghetti P., Cilurzo F. (2022). Design of Liposomal Lidocaine/Cannabidiol
Fixed Combinations
for Local Neuropathic Pain Treatment. Pharmaceutics.

[100] Franzè S., Ricci C., Del Favero E., Rama F., Casiraghi A., Cilurzo F. (2023). Micelles-in-Liposome
Systems Obtained by Proliposomal Approach for Cannabidiol Delivery:
Structural Features and Skin Penetration. Mol.
Pharmaceutics.

[101] Tiboni M., Tiboni M., Pierro A., Del Papa M., Sparaventi S., Cespi M., Casettari L. (2021). Microfluidics
for Nanomedicines Manufacturing: An Affordable and Low-Cost 3D Printing
Approach. Int. J. Pharm..

